# Soluble CD46 as a diagnostic marker of hepatic steatosis

**DOI:** 10.1016/j.ebiom.2024.105184

**Published:** 2024-06-04

**Authors:** Florian Bitterer, Paul Kupke, Akinbami Adenugba, Katja Evert, Gunther Glehr, Paloma Riquelme, Lena Scheibert, Giulia Preverin, Christina Böhm, Matthias Hornung, Hans J. Schlitt, Jürgen J. Wenzel, Edward K. Geissler, Niloufar Safinia, James A. Hutchinson, Jens M. Werner

**Affiliations:** a Department of Surgery, University Hospital Regensburg, Regensburg 93053, Germany; b Institute of Pathology, University of Regensburg, Regensburg 93053, Germany; c Institute of Clinical Microbiology and Hygiene, University of Regensburg, Regensburg 93053, Germany; d Oxford Nanopore Technologies PLC, Oxford Science Park, Oxford OX4 4DQ, United Kingdom; e Department of Hepatology, King’s College London, London SE5 8AF, United Kingdom

**Keywords:** Hepatic steatosis, Fatty liver disease, Predictive marker, Innate immunity

## Abstract

**Background:**

The increasing prevalence of metabolic dysfunction-associated steatotic liver disease (MASLD) incurs substantial morbidity, mortality and healthcare costs. Detection and clinical intervention at early stages of disease improves prognosis; however, we are currently limited by a lack of reliable diagnostic tests for population screening and monitoring responses to therapy. To address this unmet need, we investigated human invariant Natural Killer T cell (iNKT) activation by fat-loaded hepatocytes, leading to the discovery that circulating soluble CD46 (sCD46) levels accurately predict hepatic steatosis.

**Methods:**

sCD46 in plasma was measured using a newly developed immuno-competition assay in two independent cohorts: Prospective living liver donors (n = 156; male = 66, female = 90) and patients with liver tumours (n = 91; male = 58, female = 33). sCD46 levels were statistically evaluated as a predictor of hepatic steatosis.

**Findings:**

Interleukin-4-secreting (IL-4^+^) iNKT cells were over-represented amongst intrahepatic lymphocytes isolated from resected human liver samples. IL-4^+^ iNKT cells preferentially developed in cocultures with a fat-loaded, hepatocyte-like cell line, HepaRG. This was attributed to induction of matrix metalloproteases (MMP) in fat-loaded HepaRG cells and primary human liver organoids, which led to indiscriminate cleavage of immune receptors. Loss of cell-surface CD46 resulted in unrepressed differentiation of IL-4^+^ iNKT cells. sCD46 levels were elevated in patients with hepatic steatosis. Discriminatory cut-off values for plasma sCD46 were found that accurately classified patients according to histological steatosis grade.

**Interpretation:**

sCD46 is a reliable clinical marker of hepatic steatosis, which can be conveniently and non-invasively measured in serum and plasma samples, raising the possibility of using sCD46 levels as a diagnostic method for detecting or grading hepatic steatosis.

**Funding:**

F.B. was supported by the Else Kröner Foundation (Award 2016_kolleg.14). G.G. was supported by the Bristol Myers Squibb Foundation for Immuno-Oncology (Award FA-19-009). N.S. was supported by a 10.13039/100010269Wellcome Trust Fellowship (211113/A/18/Z). J.A.H. received funding from the 10.13039/501100007601European Union’s Horizon 2020 research and innovation programme (Award 860003). J.M.W. received funding from the Else Kröner Foundation (Award 2015_A10).

**Research in context**

**Evidence before this study**

Metabolic dysfunction-associated steatotic liver disease (MASLD) is a major driver for chronic liver inflammation and a societally significant challenge in modern healthcare. Since early stages of hepatic steatosis are often not reflected by specific symptoms or biochemical changes and a definitive diagnosis only succeeds through invasive interventions, the current focus lies on the development of reliable and cost-effective screening methods, including non-invasive biomarkers.

**Added value of this study**

We identified soluble CD46 (sCD46) in blood as an accurate marker of hepatic steatosis. The classification performance of sCD46 plasma levels alone was superior to previously reported composite biomarkers of hepatic steatosis.

**Implications of all the available evidence**

We conclude that non-invasive measurements of sCD46 can be a useful alternative or complement to current diagnostic methods for detecting or grading hepatic steatosis, both in routine practice and clinical trials.

## Introduction

Worldwide, metabolic dysfunction-associated steatotic liver disease (MASLD) has become one of the most common causes of chronic liver disease, affecting a quarter of the global population.^1^ MASLD begins with excessive deposition of triglycerides in the liver, which form lipid droplets within hepatocytes, a pathological condition known as hepatic steatosis. Development of steatosis reflects a chronic imbalance between, on the one hand, hepatic fatty acid uptake and triglyceride synthesis, and on the other, triglyceride metabolism and excretion.^2^ Steatosis itself is not harmful and can usually be reversed by treating the underlying cause through lifestyle modifications and tighter glycaemic control.^3^^,^^4^ However, if untreated, triglyceride deposition causes oxidative stress to hepatocytes, that eventually leads to chronic inflammation of the liver.^5^^,^^6^

MASLD encompasses a range of histopathological abnormalities, from benign steatosis to steatohepatitis and cirrhosis.^7^ The prevalence of MASLD is equal in men and women, typically presenting in the 4th or 5th decade of life, with increasing numbers in children and adolescents.^8^ Early symptoms of steatotic liver disease may include fatigue or abdominal discomfort; however, clinical suspicion is more commonly triggered by incidental discovery of hepatomegaly, biochemical disturbances or radiological signs.^9^ Typical biochemical changes include mildly or moderately elevated aminotransferases, γ-glutamyltransferase and ferritin levels, as well as disturbed amino acid and lipid metabolism.^10^ Because these features are not definitive biomarkers, diagnosis usually depends upon abdominal ultrasound or magnetic resonance-based techniques.^11^ Imaging alone cannot distinguish benign fat deposition from steatohepatitis; therefore, for routine diagnosis and follow-up of adult patients with MASLD, national guidelines recommend that liver biopsy is only performed to confirm the presence or absence of fibrosis when imaging is inconclusive.^12^^,^^13^

Early detection of steatotic liver disease leading to earlier intervention might improve prognosis. Unfortunately, with our current reliance upon imaging, we lack cost-effective screening methods to identify patients with steatosis, even within high-risk populations.^14^ Various non-invasive, composite biomarkers for hepatic steatosis have been described, including the Fatty Liver Index (FLI).^15^^,^^16^ Although these clinical scores are widely used in research and routine practice, it would be ideal to find a single, easily interpretable blood marker with a high discriminatory capacity that could be cost-effectively used for diagnosis, screening or monitoring treatment responses.^17^^,^^18^

The precise sequence of events that connects lipid-induced stress in hepatocytes and liver inflammation is not fully resolved.^19^ However, innate immunity has emerged as a critical driver of progression from hepatic steatosis to inflammation.^20^^,^^21^ The liver is densely populated by innate-like lymphocytes, including Natural Killer (NK) cells, Natural Killer T (NKT) cells and Mucosal-associated Invariant T (MAIT) cells.^22^^,^^23^ Of special interest, NKT cells are a minor subpopulation of TCRαβ-expressing T cells that respond to glycolipids presented by CD1d.^24^ NKT cells are classified as Type I (invariant) and Type II NKT cells.^25^ Human invariant Natural Killer T cells (iNKT) are characterised by co-expression of classic NK cell markers and the invariant TCR-Vα24-Jα18 chain, often in conjunction with TCR-Vβ11, which allows them to recognise α-galactosylceramide (α-GalCer) in the context of CD1d. An essential feature of iNKT cells is their ability to respond rapidly to pathogenic insults by secreting high levels of cytokines.^26^

Others previously found that a prolonged high-fat diet expands intrahepatic interleukin-4 (IL-4)-producing iNKT cells in mice.^27^ In this study, we confirmed an over-representation of IL-4^+^ iNKT cells amongst intrahepatic lymphocytes (IHL) of patients with steatosis, suggesting an unexplained role for IL-4^+^ iNKT cells in the immunopathology of steatohepatitis. *In vitro* experiments attributed this effect to enzymatic degradation of CD46, a cell-surface receptor expressed by hepatocytes to control IL-4^+^ iNKT cell differentiation. Looking for evidence of CD46 cleavage in patients, we found that soluble CD46 (sCD46) levels in blood accurately predicted steatosis grade. We conclude that sCD46 is a promising non-invasive marker for hepatic steatosis.

## Methods

### Study approval and patient cohorts

**Fig. 1:**
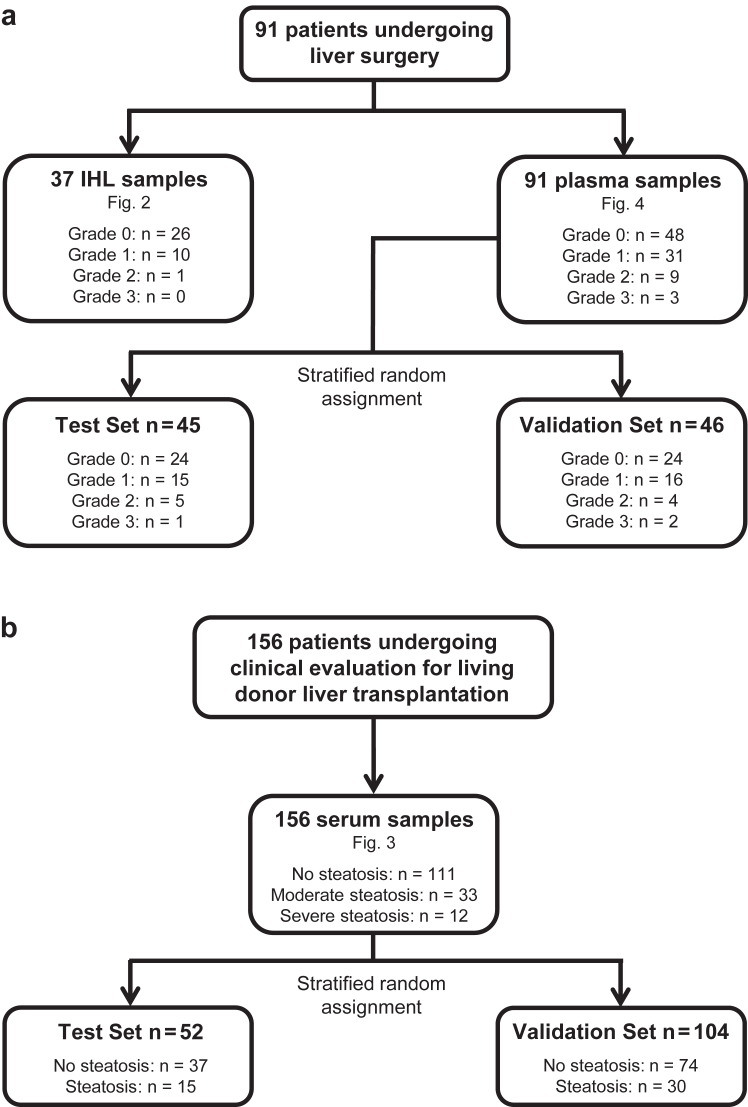
**Recruitment of the patient cohorts and allocation to the training and validation sets. (a)** Clinical samples were obtained from 91 patients undergoing liver surgery for primary liver malignancies (hepatocellular or cholangiocellular carcinoma) or benign liver lesions. This single-centre, prospective, non-randomised, observational study was authorised by the Ethics Committee of the University of Regensburg (approval number 13-257-101) and registered with clinicaltrials.gov (NCT04943978). All participants gave full, informed written consent. The first reported patient was recruited in August 2014 and the last reported patient was recruited in September 2019. Patients received standard-of-care treatment according to local guidelines. **(b)** Bio-banked samples were obtained from 156 healthy individuals, who were undergoing clinical evaluation to become living liver transplant donors. This single-centre, observational study was authorised by the Ethics Committee of the University of Regensburg (approval 13-257-101).

Here, we report outcomes from two independent cohorts of patients recruited and treated at University Hospital Regensburg in Germany (Fig. 1). Patients from study 1 (n = 156) were healthy individuals undergoing clinical evaluation to become living liver transplant donors, who were assigned to “training” and “validation” sets; patients from study 2 (n = 91) underwent partial liver resection for hepatic tumour, and were also distributed into “training” and “validation” sets. Bio-banked samples were obtained from study 1 participants, who were included in an observational study that conformed to all applicable laws and ethical standards (Fig. 1A; Supplementary Fig. S1). The study was authorised by the Ethics Committee of the University of Regensburg (13–257_5-101). Study 2 patients were recruited to a single-centre, prospective, observational study that conformed to all applicable laws and ethical standards, including the Declaration of Helsinki (Fig. 1A; Supplementary Fig. S2). The study was authorised by the Ethics Committee of the University of Regensburg (13-257-101) and registered with clinicaltrials.gov (NCT04943978). All study 2 participants gave full, informed written consent. The first reported patient was recruited in August 2014 and the last reported patient was recruited in September 2019. All study 2 patients received standard-of-care treatment according to local guidelines; notably, no patients received systemic chemotherapy before surgery. Clinical and demographic data, including information about sex, age and race, were taken from hospital records. Waist circumference was measured from abdominal CT images according to established standards.

### Sonographic classification of steatosis

During the evaluation for living liver transplant donation, upper abdominal sonography was performed on each patient. This involved routine characterisation of the liver parenchyma. The degree of steatosis was determined by the ratio of the echogenicity of liver and kidney. The lighter the parenchyma of the liver compared to the kidney, the higher the degree of steatosis classified by the examiner.

### Histopathological examination of human liver specimens

Liver tissue specimens were fixed in neutral buffered formalin before embedding in paraffin. 4 μm thick sections were prepared, deparaffinised with ethanol and xylene, and then stained with Haematoxylin and Eosin (HE) and Elastica van Gieson (EvG) or Sirius Red. Staining with EvG or Sirius Red was used to evaluate liver fibrosis according to the Ishak scoring system.^28^ Steatosis of the liver was reported as percentage of hepatocytes containing fat droplets. By convention, patients with <5% steatosis were considered to be non-steatotic. Grade 1 steatosis was diagnosed at a minimum of 5%. From 34% to 67%, steatosis was defined as grade 2 or 3, respectively.^29^

### Processing of PBMC and IHL from clinical material

Human intrahepatic lymphocytes (IHL) were isolated as previously described.^30^ Briefly, resected liver tissue was verified as tumour-free by a pathologist, then was washed with HBSS (Sigma–Aldrich) medium before dissection into small fragments. After digestion with 0.5 mg/ml collagenase type IV (Merck) and 50 ng/ml DNase I (Applichem), hepatocytes were removed by filtration through a 40 μm mesh (Greiner). IHL were then collected by Ficoll gradient centrifugation.

Human iNKT cells and iNKT-depleted peripheral blood mononuclear cells (PBMC) were obtained from leukoreduction chambers of Trima apheresis device as a by-product of thrombocyte donation. Leucocytes were separated from cell suspensions by Ficoll density gradient centrifugation and the isolated cells were frozen in RPMI (Gibco) containing 10% dimethyl sulfoxide (DMSO; AppliChem) under liquid nitrogen. Cryovials were thawed at 37 °C before transferring the cell suspension to prewarmed RPMI containing 100 μg/ml DNase I.

### Isolation, expansion and treatment of iNKT cells

Vα24-Jα18^+^ iNKT cells were magnetically isolated from PBMC using anti-iNKT microbeads (Miltenyi) with the program POSSEL_S on an AutoMACS Pro device (Miltenyi). iNKT expansion medium comprised RPMI 1640 GlutaMAX™, 10% HyClone FetalClone II serum (GE Healthcare), 1% sodium pyruvate (Gibco), 1% MEM non-essential amino acids (Gibco), 7.5% NaHCO_3_ (Gibco), 1% penicillin/streptomycin (Sigma–Aldrich) and 50 μM 2-mercaptoethanol (Sigma–Aldrich). Enriched iNKT cells were seeded at 5·10^4^ cells/cm^2^ in iNKT medium supplemented with 50 ng/ml of animal-free rhIL-2 (Peprotech) and 100 ng/ml α-Galactosylceramide (GalCer; Biomol) before expansion for 7 d. The medium was replaced on day 2 with iNKT medium supplemented with rhIL-2 and α-GalCer. On day 5, the medium was replaced with iNKT medium supplemented with rhIL-2 only. Treatment of *ex vivo*-expanded iNKT cells with chimeric CD46-Fc (10257-CD; R&D Systems) or control Fc protein was renewed with each medium change.

### Differentiation, fat loading (FL) and manipulation of HepaRG cells

HepaRG cells (RRID: CVCL_9720) were purchased from Biopredic International, France, and routinely tested for contamination with mycoplasma. For two weeks, HepaRG cells were cultured in 12-well plates or T75 flasks (TPP) at 2.5·10^4^ cells/cm^2^ in HepaRG growth medium comprising William’s E medium (Gibco) supplemented with 10% HyClone FetalClone II serum, 1% penicillin/streptomycin, 1% L-glutamine (Sigma–Aldrich), 0.023 IU/ml insulin (Lilly), 4.7 μg/ml hydrocortisone (Pfizer) and 80 μg/ml gentamycin (Rotexmedica). HepaRG cells were then cultured for a further 2 weeks in HepaRG growth medium supplemented with 1.8% DMSO to aid differentiation into hepatocyte-like cells. Differentiated HepaRG cells were cultured for 24 h in serum-free HepaRG growth medium and then treated with bovine serum albumin (BSA) conjugated-palmitic acid and oleic acid (1:2–0.5 mM; Sigma–Aldrich) dissolved in isopropanol (Sigma–Aldrich) for a further 24 h to induce fat loading (FL) of HepaRG cells (FL-HepaRG). We used unloaded (UL)-HepaRG treated with isopropanol as vehicle-only control cells. Fat loading of HepaRG cells was confirmed by Oil Red O staining (ScienCell) according to the manufacturer’s instructions. For flow cytometry analysis, adherent HepaRG cells were detached from plastic culture surfaces with Cell Staining Buffer (BioLegend) and mechanical scraping.

### iNKT cell and HepaRG cell cocultures

To investigate the interaction of iNKT cells and HepaRG cells *in vitro*, coculture experiments were performed. 2·10^5^ freshly sorted iNKT cells were added to differentiated UL- or FL-HepaRG cells in 12-well plates with iNKT expansion medium, which was changed after 4 days. Indirect coculture experiments were performed with 0.4 μm transwell inserts (Corning). After 7 days’ coculture, the non-adherent iNKT cells were harvested and further analysed. In some experiments, α-CD46 (AF2005; R&D) or goat Ig control were added to freshly isolated iNKT cells before coculture.

### Isolation, differentiation and fat loading of primary human liver organoids

Primary human liver organoids were generated using protocols adapted from the literature.^31^^,^^32^ Our various cell culture media are described as Supplementary Materials (Supplementary Fig. S3). Briefly, ∼1 g surgically resected liver tissue was minced into ∼1 mm^3^ pieces using scissors and scalpel. After washing, the liver fragments were incubated in digestion medium at 37 °C for at least 30 min. Afterwards the cell suspension was filtered, washed and resuspended in basement matrix extract (Cultrex® Basement Membrane Extract, Type 2; BME2; Merck) before single droplets were transferred into culture plates. When solidified, isolation medium was added. 3–4 d later, the developing organoids were switched into expansion medium. From that point on, medium was renewed every 3–4 d until sufficient expansion was achieved. Typically, organoids were recognizable during the first week. To induce differentiation, 100 ng/ml BMP7 (PeproTech) was added to the expansion medium for 2 d. Subsequently, we produced cell monolayers by resuspending organoid fragments in differentiation medium containing 5% BME2. To induce fat loading, organoids were cultured for 12 d in differentiation medium, which was renewed at 3–4 d intervals. Organoids were then “starved” in diet medium for 24 h before being treated with fat loading medium for a further 24 h. Unloaded (vehicle-only) control organoids were generated using isopropanol, which was used as a solvent for the fatty acids.

### Flow cytometry

Antibody panels and gating strategies are specified in the Supplementary Figs. S4 and S5. As previously described, cell-surface staining was performed at 4 °C in Cell Staining Buffer with 10% FcR-block (Miltenyi) for 30 min.^33^ The FoxP3 Fixation-and-Permeabilization Kit (eBioscience) was used according to the manufacturer’s instructions for intracellular staining. Dead cells were excluded with Fixable Viability Dye eFluor506 (Invitrogen) or ViaKr-808 (Beckman Coulter). For intracellular detection of cytokines, cultured iNKT cells were harvested, washed and then stimulated with 50 ng/ml PMA and 1 μg/ml ionomycin in the presence of brefeldin A and monensin (all from BD Biosciences) for 4 h prior to staining.^33^ Data were collected with a Navios or CytoFlex LX cytometer, and were analysed with Kaluza 2.1 (all from Beckman Coulter). Marker expression was estimated as background (isotype)-subtracted mean fluorescence intensities (MFI). Background-subtracted MFI values ≤ 0 were set to 1.

### Measurement of sCD46 concentration

**Fig. 2:**
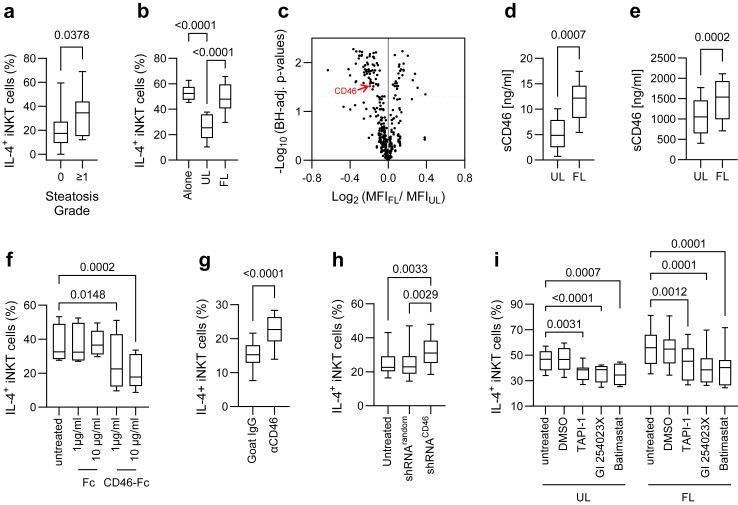
**Discovery of soluble CD46 as a marker of steatotic human hepatocytes. (a)** Frequency of intrahepatic IL-4^+^ iNKT cells with respect to iNKT cells isolated from normal human liver tissue [Grade 0, n = 26; Grade ≥1, n = 11; Wilcoxon rank-sum test after Shapiro–Wilk test was significant (p = 0.0336)]. **(b)** Frequency of IL-4^+^ iNKT cells after 7 days’ expansion, alone or in coculture with unloaded (UL)- or fat-loaded (FL)- HepaRG cells [n = 8; one-way repeated measures ANOVA p < 0.0001; post-hoc paired t-test with BH-adjusted p-values]. **(c)** Flow cytometric expression analysis of 369 surface markers on paired UL- and FL-HepaRG cell cultures [n = 6; paired t-test with BH-adjusted p-values]. **(d)** Concentration of sCD46 in supernatants from UL- and FL-HepaRG cultures measured with a competitive cell-based flow cytometry assay [n = 20; unpaired t-test]. **(e)** Concentration of sCD46 in supernatants from UL- and FL-primary human liver organoid cultures measured by ELISA [n = 8; paired t-test]. **(f)** Frequency of IL-4^+^ iNKT cells after 7 days’ expansion in the absence or presence of recombinant human CD46-Fc or control Fc protein [n = 6, one-way repeated measures ANOVA p = 0.0008; post-hoc paired t-test with BH-adjusted p-values]. **(g)** Frequency of IL-4^+^ iNKT cells after 7 days’ expansion in UL-HepaRG cell cocultures treated with of 5 μg/ml neutralizing α-CD46 or isotype-control antibody [n = 14; paired t-test]. **(h)** Frequency of IL-4^+^ iNKT cells after 7 days’ expansion in coculture with UL-HepaRG cells stably transfected with random-sequence shRNA or CD46 shRNA. Untransfected UL-HepaRG cells were used as controls [n = 15; one-way repeated measures ANOVA p = 0.0007; post-hoc paired t-test with BH-adjusted p-values]. **(i)** Frequency of IL-4^+^ iNKT cells after 7 days’ expansion in coculture with UL- or FL-HepaRG cells that were untreated or treated with 10 μM MMP inhibitor (TAPI-1, GI254023X and Batimastat) or DMSO [n = 8; two-way repeated measures ANOVA (p treatment < 0.0001, p loading = 0.043); post-hoc paired t-test with BH-adjusted p-values].

sCD46 levels in culture supernatants and plasma samples were measured using a flow cytometry-based competition assay in all experiments, except those shown in Fig. 2E. A step-by-step protocol is given as Supplementary Material (Supplementary Fig. S6). Briefly, 3·10^5^ CD46-expressing MOLT-4 cells (RRID: CVCL_0013) were suspended in a final reaction volume of 100 μl comprising Cell Staining Buffer, 0.25 μl α-CD46-PE mAb (130-104-508; Miltenyi) plus test sample or rhCD46 calibration controls. The presence of competing sCD46 in samples reduced binding of α-CD46 to MOLT-4 cells, allowing the calculation of absolute concentrations using calibration controls. For Fig. 2E, we measured release of sCD46 by primary human liver organoids using a sandwich ELISA. Standard polystyrene microplates (DY990; R&D) were coated with anti-human CD46 antibody (MAB2005; R&D) in phosphate buffered saline (PBS; Gibco) at 4 μg/ml for 24 h. Wells were then blocked for 1 h using reagent diluent (DY995; R&D) and washed extensively with wash buffer (WA126; R&D) before applying calibration and test samples. Starting at 2 ng/ml, doubling dilutions of rhCD46 His-tag protein (10256-CD; R&D) were used to calibrate measurements. An anti-human CD46-biotin antibody (BAF2005; R&D) was used for detection at 50 ng/ml for 2 h, followed by streptavidin-HRP (DY998; R&D) for 20 min.

**Fig. 3:**
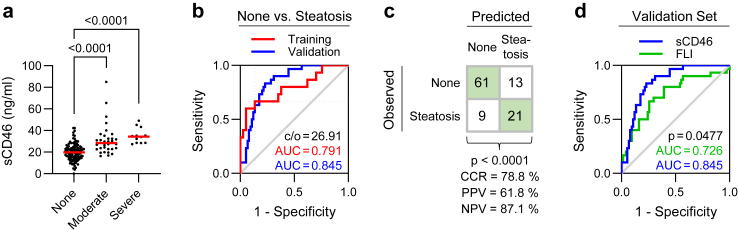
**Soluble CD46 levels accurately predict hepatic steatosis. (a)** sCD46 levels in serum samples from prospective living liver transplant donors with varying degrees of steatosis, which was assessed by ultrasononography [none, n = 111; moderate, n = 33; severe, n = 12; one-way ANOVA p < 0.0001; post-hoc unpaired t-test with BH-adjusted p-values]. **(b)** Receiver Operating Characteristic (ROC) curves illustrating the capacity of sCD46 levels to discriminate between patients with or without steatosis detected by ultrasonography. Patients were assigned to training (n = 52) or validation (n = 104) sets by stratified randomization. A discriminatory cut-off for sCD46 of 26.91 ng/ml was established from training set data (dotted lines) using the smallest distance to top left. **(c)** Cross-tabulation of predicted and observed steatosis in validation set patients using a sCD46 cut-off value of 26.91 ng/ml [n = 104; Fisher’s exact test]. **(d)** ROC curves comparing the performance of sCD46 serum levels and FLI as predictors of hepatic steatosis in validation set patients [n = 104; one-sided DeLong test].

### Stable knockdown of CD46 in HepaRG cells

CD46 and scrambled sequence shRNA plasmids were obtained from Santa Cruz (sc-35004-SH). HepaRG cells were transfected using Lipofectamine 3000 (ThermoFisher) according to the manufacturer’s instructions before selection with 1.5 μg/ml puromycin (Santa Cruz). CD46^low^ stable transfectants were sorted to purity using a BD FACSAria™ II.

### qRT-PCR for CD46 isoforms

RNA isolation and reverse transcription from UL- and FL-HepaRG cells was performed using an RNeasy Mini Kit (Qiagen) and SuperScript™ III (Invitrogen). Real-time PCR for CD46 transcript variants was adapted from a method published by Hansen et al.^34^ LightCycler® 480 Probes Master (Roche) and a Roche LightCycler 480 were used to determine the relative proportion of the four different CD46 isoforms (B1, C1, BC1, BC2). Primer and probe sequences, and their respective cycling concentrations are given in Supplementary Fig. S7.

### Statistics

Significance tests and generating ROC curves were performed in GraphPad Prism, SPSS® or R (4.3.3). As indicated in figure legends, significance tests were performed with the Wilcoxon rank-sum test, paired and unpaired t-tests, one-way and two-way ANOVA, or DeLong test. Where appropriate, p-values were adjusted for multiple comparison. Only significant p-values are shown within the figures. We generally use a significance level of 0.05.

For area under the ROC curves and confidence intervals of all performance measures we used the pROC package (1.18.5) and applied bootstrapping with 10,000 iteration and a confidence level of 95%. For the training sets, we report the performances recalculating the ROC curve on the bootstrap. In contrast, for the validation sets we use the fixed cutoff from the respective training set and then calculate the performance measures on the bootstraps.

We used the DeLong test^35^ to compare ROC curves with the pROC package implementing a recent fast algorithm.^36^

### Role of funders

Funding agencies were not involved in study design, data collection, data analyses, interpretation, or writing of this report.

## Results

### Identifying soluble CD46 as a marker of steatotic human hepatocytes

IL-4^+^ iNKT cells were over-represented amongst intrahepatic lymphocytes (IHL) from patients with steatosis undergoing partial liver resection (Fig. 1A; Fig. 2A). To explain this difference, we next investigated *in vitro* reactions of human iNKT cells to a hepatocyte-like cell line (HepaRG) after fat loading (Supplementary Fig. S4A and B). When isolated iNKT cells were expanded for 7 days using recombinant human (rh) IL-2 and α-GalCer in direct contact with fat-loaded (FL)-HepaRG cells, a higher proportion of IL-4^+^ iNKT cells was detected than in cocultures with unloaded (UL)-HepaRG cells (Fig. 2B). Other cytokine-producing iNKT cell subsets were unaffected (Supplementary Fig. S4C–E). Comparing iNKT cells expanded alone or in coculture revealed an impaired capacity of FL-HepaRG cells to suppress IL-4^+^ iNKT cell development, which was a contact dependent effect (Fig. 2B; Supplementary Fig. S4F). These results implied the presence of a cell-surface ligand on HepaRG cells that interacts with iNKT cells to inhibit IL-4 expression, but not with conventional T cells (Supplementary Fig. S4G). To identify this putative ligand, we screened for differentially expressed cell-surface antigens in FL- and UL-HepaRG cells by flow cytometry (Fig. 2C). This returned 67 significantly down-regulated receptors, including CD46, which was not differentially expressed at the mRNA level, implying post-translational regulation (Supplementary Fig. S7).

A soluble form of CD46 (sCD46) was detected at higher levels in FL-HepaRG cultures compared to UL controls (Fig. 2D). Likewise, sCD46 levels were higher in supernatants of fat-loaded primary human liver organoids compared to vehicle-only control cultures (Fig. 2E). Adding chimeric CD46-Ig protein to isolated iNKT cells suppressed IL-4 expression (Fig. 2F). In coculture, blocking CD46 with neutralising antibodies or silencing *CD46* mRNA in HepaRG cells promoted IL-4^+^ iNKT cell development (Fig. 2G&H; Supplementary Figs. S5 and S8). Expression of matrix metalloproteinases (MMP)-1, −3, −7 and −10 was significantly higher in FL-HepaRG cocultures (Supplementary Fig. S9A). Applying MMP-inhibitors caused a dose-dependent increase in cell-surface CD46 expression and corresponding reduction in IL-4^+^ iNKT cells (Fig. 2I; Supplementary Fig. S9B). Hence, we found that fat-induced degradation of cell-surface CD46 by MMPs leads to disinhibited IL-4^+^ iNKT cell differentiation.

### Confirming sCD46 as a diagnostic marker of steatosis

To establish the *in vivo* relevance of our discovery, we obtained samples from a test cohort of n = 156 prospective living-donor liver transplant donors, who were healthy individuals undergoing assessment for partial hepatectomy (Fig. 1B; Supplementary Fig. S1). In this cohort, steatosis was assessed by ultrasonography following national guidelines.^12^ Individuals with moderate or severe steatosis exhibited higher sCD46 levels than those without detectable steatosis (abbreviated to ‘none’) (Fig. 3A). After stratified random assignment to a training set (n = 52) and a validation set (n = 104) that ensured equal representation of all steatosis grades in both groups, we found that sCD46 performed well in discriminating between non-steatotic and steatotic individuals (Fig. 3B). Training set data were used to determine a cut-off for sCD46 of 26.91 ng/ml that discriminated between non-steatotic and steatotic patients (Supplementary Fig. S11A). In the validation set, the correct classification rate (CCR) was 78.8%, positive predictive value (PPV) was 61.8%, and negative predictive value (NPV) was 87.1% (Fig. 3C).

To understand the potential clinical impact of sCD46 as a marker of steatosis, we compared its performance with 5 composite markers – namely, Fatty Liver Index (FLI), Lipid Accumulation Product (LAP), Visceral Adiposity Index (VAI), Triglyceride Glucose Index (TyG) and Hepatic Steatosis Index (HSI).^37^, ^38^, ^39^, ^40^ Of these established scores, FLI performed best in our transplant donor cohort (Supplementary Fig. S10). As a single feature, sCD46 was a better discriminator than FLI, a 4-factor model (Fig. 3D; Supplementary Fig. S11). We conclude that, in our dataset, sCD46 is superior to established composite clinical markers of steatosis.

### Soluble CD46 levels accurately predict hepatic steatosis

**Fig. 4:**
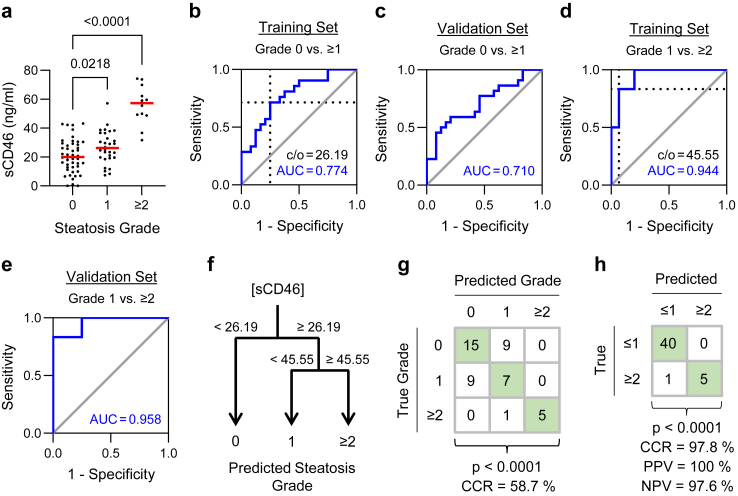
**Confirming sCD46 as a diagnostic marker of steatosis. (a)** Distribution of sCD46 concentration in plasma samples from patients with varying degrees of steatosis evaluated by histology [Grade 0, n = 48; Grade 1, n = 31; Grade ≥2, n = 12; one-way ANOVA p < 0.0001; post-hoc unpaired t-test with BH-adjusted p-values]. **(b)** Receiver Operating Characteristic (ROC) curve illustrating the capacity of sCD46 levels to discriminate between Grade 0 and Grade ≥1 steatosis in training set patients (n = 45). An optimal cut-off value for sCD46 of 26.19 ng/ml was set using the smallest distance to top left (dotted lines). **(c)** ROC curve discriminating Grade 0 and Grade ≥1 steatosis in validation set patients (n = 46). **(d)** ROC curve discriminating Grade 1 and Grade ≥2 steatosis in training set patients (n = 21). An optimal cut-off value for sCD46 of 45.55 ng/ml was set using the smallest distance to top left (dotted lines). **(e)** ROC curve discriminating Grade 1 and Grade ≥2 steatosis in validation set patients (n = 22). **(f)** A decision tree for predicting steatosis grade based upon plasma sCD46 levels. **(g)** Cross-tabulation of predicted steatosis grade in validation set patients [n = 46; Fisher’s exact test]. **(h)** Cross-tabulation of predicted steatosis grade in validation set patients considering only the most clinically relevant distinction between Grade ≤1 and Grade ≥2 steatosis [n = 46; Fisher’s exact test].

To rigorously evaluate sCD46 as a diagnostic marker of steatosis, we next measured sCD46 levels in n = 91 plasma samples from patients with liver tumours (Fig. 4A). Samples were assigned by stratified randomization to a training (n = 45) or validation (n = 46) set ensuring equal representation of steatosis cases in both groups (Fig. 1A). In this cohort, steatosis was assessed by histology instead of ultrasonography. Broadly speaking, a histological grade of ≤1 corresponds to an ultrasound diagnosis of no detectable steatosis; however, agreement between these methods is imperfect owing partly to operator dependency. Accordingly, we calculated a new discriminatory cut-off value for sCD46 using histological grading as the response variable. Training set data were used to determine a cut-off for sCD46 of 26.19 ng/ml that discriminated between patients with no histologically evident steatosis (Grade 0) and patients with any degree (Grade ≥1) of steatosis (Fig. 4B; Supplementary Fig. S12). The discriminatory value of sCD46 was confirmed in the validation set (Fig. 4C).

Next, we asked whether sCD46 distinguished between benign steatosis (Grade 1) and more extensive fat deposition (Grade ≥2). We found sCD46 levels also reliably discriminated between steatosis Grade 1 and ≥ 2 in the training and validation sets using a second sCD46 cut-off value of 45.55 ng/ml (Fig. 4D and E; Supplementary Fig. S13). Accordingly, we constructed a decision tree to predict steatosis grade in patients (Fig. 4F) with an overall correct prediction rate of 58.7% in the validation set (Fig. 4G). Collapsing this model to classify patients as either having Grade ≤1 or Grade ≥2 steatosis resulted in excellent predictive performance (Fig. 4H; Supplementary Fig. S14). In the validation set, the correct classification rate (CCR) was 97.8%, positive predictive value (PPV) was 100%, and negative predictive value (NPV) was 97.6%.

## Discussion

There have been intense efforts to develop predictive biomarkers for hepatic steatosis, inflammation and fibrosis. Although liver biopsy remains a valuable tool for cases of diagnostic uncertainty and, at present, is still required in late-phase clinical trials, non-invasive biomarkers have already largely replaced biopsies in routine assessment of inflammation or fibrosis risk in MASLD patients.^41^ Our discovery of sCD46 as a promising biomarker of hepatic steatosis, as opposed to inflammation or fibrosis, complements the existing toolbox of predictive biomarkers.

In two independent cohorts, hepatic steatosis was reliably predicted by sCD46 levels and, importantly, performed better than common diagnostic scores for hepatic steatosis. It is uncomplicated to measure sCD46 in serum and plasma samples by immunocompetition assay or ELISA, so sCD46 might be a valuable alternative to current non-invasive methods for detecting or grading hepatic steatosis.^12^ As a screening tool, we demonstrated that sCD46 accurately classifies patients with or without steatosis amongst cohorts that were not purposely selected for risk of MASLD. We imagine that sCD46 could be useful for preselecting middle-aged adults who might benefit from assessing their risk of disease progression by histology, vibration-controlled transient elastography, Fibrosis-4 Index (FIB-4), NAFLD Fibrosis Score (NFS), Enhanced Liver Fibrosis (ELF™) score, AST to platelet ratio index (APRI) or FibroTest®.^42^, ^43^, ^44^, ^45^, ^46^, ^47^ Although not investigated as part of this study, sCD46 might be a convenient analyte for tracking responses to therapy in patients under care of steatotic liver outpatient clinics or participating in trials.

Previous attempts to discover serum protein biomarkers of steatosis did not find sCD46, but returned other predictive markers. Notably, a proteomic screening of samples from 69 MASLD patients and 16 healthy controls identified 605 differentially represented proteins in serum. Two of these proteins, prothrombin fragment and paraoxonase 1, discriminated steatotic liver disease patients from controls with high accuracy in training data.^48^ Subsequent studies supported the predictive value of lowered serum paraoxonase 1 as a biomarker of steatosis.^49^^,^^50^ Elsewhere, pentraxin 3 (PTX3) and squalene epoxidase (SQLE) levels in blood discriminated patients with simple steatosis from those with steatohepatitis.^51^^,^^52^ We must perform larger studies to know whether a composite biomarker panel including sCD46 and these other proteins improves their overall predictive performance.

In our cohorts, sCD46 performed particularly well as a biomarker of steatosis because expression was relatively consistent in non-steatotic patients; therefore, we could reliably detect small changes in sCD46 levels. Other conditions that disturb sCD46 levels in blood could affect our interpretation of sCD46 as a biomarker of hepatic steatosis. CD46 expression is upregulated in hepatocellular carcinoma (HCC) and other malignancies, possibly to evade complement-dependent cytotoxicity, but it is unknown whether circulating sCD46 levels are correspondingly increased.^53^ Elevated sCD46 levels were observed in patients with multiple sclerosis (MS) and systemic lupus erythematosus (SLE), likely as consequence of MMP9-mediated shedding from activated T cells.^54^^,^^55^ Clearly, in future studies, we must be mindful of alternative explanations when interpreting sCD46 as a marker of steatosis.

Our clinical interpretation of sCD46 serum and plasma levels is shaped by our basic immunological discoveries that link fat loading of hepatocytes to activation of innate immune cells. In our proposed model, fat-induced stress in hepatocytes leads to up-regulation of MMP secretion, which in turn leads to non-specific cleavage of CD46 from the hepatocyte surface. The functional consequence, at least for iNKT cells, is an overall disinhibition that permits IL-4 expression.^56^ The immunopathological relevance of human IL-4^+^ iNKT cells in steatotic liver disease is not presently known. In mice, iNKT cell-derived IL-4 exacerbates liver inflammation in some circumstances^57^^,^^58^ but suppresses it in others.^59^, ^60^, ^61^ A deeper understanding of the role of IL-4^+^ iNKT cells in liver disease might lead to a more refined interpretation of sCD46 as a biomarker. At present, it is not known whether this mechanism is specific to fat loading of hepatocytes in MASLD patients. It’s a limitation of our study that sCD46 levels were not assessed in patients with other aetiologies, such as alcohol-related liver disease or chemotherapy-associated steatohepatitis.

Shedding of CD46 from hepatocytes into circulation reflects a stress response of hepatocytes to fat loading, which appears to be connected with activation of innate-like lymphocyte responses; therefore, sCD46 is unlike other liver markers that indicate cell injury, disturbed biochemical activity, fibrosis or systemic inflammation. Hence, we conclude that sCD46 is a promising clinical marker of patients with steatosis at risk of developing early liver inflammation, a subset that could benefit from earlier clinical detection and intervention.

## Contributors

F.B. and P.K. designed and performed experiments, analysed data and drafted figures; A.A. designed and performed experiments, and analysed data; K.E. performed histopathological assessment of liver biopsies; G.G. performed statistical analyses; P.R. performed experiments and edited the manuscript; L.S. extracted and collated clinical information; G.P. performed experiments; C.B. and J.J.W. provided clinical samples; M.H., H.J.S. and E.K.G. provided resources, data interpretation and critical feedback; N.S. provided expert hepatological opinion and corrected the manuscript; J.A.H. and J.M.W. conceived the study, designed experiments, analysed results, had unrestricted access to all data, and wrote the manuscript. All authors agreed to submit the manuscript, read and approved the final draft and take full responsibility for its content, including the accuracy of the data and its statistical analysis.

## Data sharing statement

All data are presented within the manuscript, Supplementary materials, accompanying Source Data file, or are available upon request from the corresponding author.

## Declaration of interests

University Hospital Regensburg has filed a not yet published European patent application (Registration Nr. 23 183 382.3) for sCD46 as a clinical biomarker of hepatic steatosis. J.A.H. received in-kind support from Beckman Coulter. The authors have no other conflicts of interest to declare.
