# Supplementary material for: Soluble CD46 as a diagnostic marker of hepatic steatosis
Source: eBioMedicine. 2024 Jun 4;104:105184. doi: 10.1016/j.ebiom.2024.105184 (PMC11179574; doi:10.1016/j.ebiom.2024.105184)
Supplement: Supplementary Figs. S1–S14 [file mmc1.pdf]

	No Steatosis	Moderate Steatosis	Severe Steatosis	p-value
**Patient Demographics**				
Included Patients (%)	111 (71.15)	33 (21.15)	12 (7.692)	
Mean Age (years) [95% CI]	36.14 [34.36-37.93]	39.73 [35.47-43.98]	42.58 [36.44-48.72]	0.5354
Sex Distribution ♂ / ♀	47 / 64	13 / 20	6 / 6	0.8293
Mean BMI (kg/m²) [95% CI]	24.35 [23.74-24.95]	27.58 [26.01-29.16]	27.18 [23.88-30.47]	**0.0002**
Diabetes Mellitus	0	0	0	
**Baseline Biochemistry**				
Mean Creatinine (mg/dl) [95% CI]	0.798 [0.771-0.824]	0.777 [0.727-0.827]	0.811 [0.686-0.936]	0.6460
Mean AST (U/l) [95% CI]	21.12 [17.80-24.44]	22.79 [17.80-27.78]	28.08 [20.49-35.68]	**0.0117**
Mean ALT (U/l) [95% CI]	25.62 [23.75-27.49]	44.33 [19.01-69.65]	35.42 [27.62-43.21]	**0.0008**
Mean ALT/AST [95% CI]	1.506 [1.346-1.666]	1.714 [1.455-1.973]	1.386 [1.023-1.750]	0.1437
Mean γGT (U/l) [95% CI]	28.03 [24.48-31.57]	34.33 [19.08-49.59]	60.67 [15.17-106.2]	**0.0106**
Mean AP (U/l) [95% CI]	71.88 [67.99-75.77]	82.91 [70.66-95.16]	72.42 [63.51-81.32]	0.1938
Mean Bilirubin (mg/dl) [95% CI]	0.601 [0.522-0.679]	0.579 [0.479-0.679]	0.571 [0.423-0.719]	0.8647
Mean Glucose (mg/dl) [95% CI]	91.30 [88.25-94.34]	91.94 [86.49-97.39]	97.75 [89.18-106.3]	0.1834
Mean Ferritin (ng/ml) [95% CI]	81.20 [67.77-94.63]	94.23 [50.64-137.8]	135.1 [73.60-196.9]	0.1566
Mean CRP (mg/l) [95% CI]	2.851 [2.579-3.123]	3.894 [2.635-5.153]	6.825 [2.759-10.89]	**0.0056**
Mean Triglycerides (mg/dl) [95% CI]	112.3 [101.9-122.6]	154.3 [120.0-188.6]	133.7 [103.9-163.4]	**0.0187**
Mean Cholesterol (mg/dl) [95% CI]	191.5 [184.9-198.1]	197.2 [183.6-210.9]	200.8 [165.3-236.2]	0.6347
Mean HDL (mg/dl) [95% CI]	56.85 [53.83-59.86]	52.18 [47.85-56.51]	50.08 [41.63-58.54]	0.1521
Mean INR [95% CI]	1.020 [1.006-1.033]	0.965 [0.943-0.987]	1.003 [0.968-1.039]	**0.0008**
Mean PTT (s) [95% CI]	30.57 [30.00-31.14]	30.09 [28.79-31.38]	28.78 [27.05-30.52]	0.0701
**Baseline Haematology**				
Mean Leucocytes (c/nl) [95% CI]	6.498 [6.197-6.800]	7.259 [6.645-7.872]	7.637 [6.766-8.508]	**0.0045**
Mean Haemoglobin (g/dl) [95% CI]	14.02 [13.77-14.26]	14.33 [13.84-14.81]	14.05 [13.19-14.91]	0.5072
Mean Thrombocytes (c/nl) [95% CI]	244.8 [235.4-254.3]	261.3 [242.0-280.6]	266.3 [213.7-318.8]	0.2625
**Steatosis Scores**				
Mean Fatty Liver Index [95% CI]	32.38 [27.95-36.81]	55.99 [45.86-66.12]	61.26 [45.86-76.66]	**<0.0001**
Mean Hepatic Steatosis Index [95% CI]	12.89 [11.57-14.21]	14.50 [12.44-16.56]	12.09 [8.730-15.45]	0.1593
Mean Lipid Accumulation Product [95% CI]	36.85 [31.99-41.71]	69.93 [50.53-89.34]	60.01 [38.67-81.35]	**<0.0001**
Mean Triglyceride-Glucose Index [95% CI]	8.418 [8.323-8.514]	8.691 [8.473-8.909]	8.719 [8.507-8.932]	**0.0183**
Mean Visceral Adiposity Index [95% CI]	1.649 [1.407-1.891]	2.579 [1.834-3.324]	2.149 [1.458-2.840]	**0.0084**

Figure S1. Characteristics of prospective living liver donors.

Clinical, biochemical and haematological characteristics of study patients according to sonographic steatosis grade. Groups were compared using Fisher's Exact Test or Kruskal-Wallis Test. p-values were not adjusted for multiple comparisons.

	Steatosis Grade 0	Steatosis Grade 1	Steatosis Grade ≥2	p-value
**Patient Demographics**				
Included Patients (%)	48 (52.75)	31 (34.07)	12 (13.19)	
Mean Age (years) [95% CI]	61.94 [57.31-66.57]	65.77 [61.36-70.19]	66.50 [59.73-73.27]	0.5354
Sex Distribution ♂ / ♀	26 / 22	23 / 8	9 / 3	0.1639
Mean BMI (kg/m^2) [95% CI]	24.62 [23.17-26.06]	28.56 [25.95-31.16]	29.04 [26.58-31.51]	**0.0007**
Diabetes Mellitus (%)	4 (8.333)	7 (22.58)	6 (50.00)	**0.0042**
**Surgery-Indicating Diagnosis**				
Benign Disease (total n = 16)	10 (62.50)	3 (18.75)	3 (18.75)	0.3453
HCC (total n = 30)	7 (23.33)	17 (56.67)	6 (20.00)	**0.0003**
CCC (total n = 45)	31 (68.89)	11 (24.44)	3 (6.667)	**0.0084**
**Baseline Biochemistry**				
Mean Creatinine (mg/dl) [95% CI]	0.807 [0.726-0.888]	0.841 [0.759-0.923]	0.829 [0.707-0.951]	0.3870
Mean AST (U/l) [95% CI]	55.32 [34.12-76.52]	36.71 [27.96-45.45]	40.91 [25.87-55.95]	0.7730
Mean ALT (U/l) [95% CI]	83.00 [41.26-124.7]	39.66 [31.62-47.69]	43.17 [32.62-53.71]	0.7173
Mean γGT (U/l) [95% CI]	322.8 [208.9-436.6]	231.4 [134.8-328.0]	154.6 [73.95-235.3]	0.5243
Mean AP (U/l) [95% CI]	243.2 [174.0-312.4]	122.0 [98.03-146.0]	112.8 [83.95-141.6]	**0.0391**
Mean Bilirubin (mg/dl) [95% CI]	1.838 [0.837-2.840]	0.758 [0.560-0.957]	0.814 [0.448-1.180]	0.6433
Mean CRP (mg/l) [95% CI]	22.72 [14.87-30.57]	26.31 [10.62-42.00]	19.60 [-4.517-43.72]	0.7240
Mean INR [95% CI]	1.007 [0.982-1.032]	1.032 [0.999-1.064]	1.031 [0.983-1.076]	0.2583
Mean PTT (s) [95% CI]	30.90 [28.75-33.04]	30.69 [28.68-32.70]	34.93 [26.13-43.72]	0.4630
**Baseline Haematology**				
Mean Leucocytes (c/nl) [95% CI]	8.120 [7.245-8.995]	8.237 [6.549-9.925]	7.016 [4.648-9.384]	0.2541
Mean Haemoglobin (g/dl) [95% CI]	12.96 [12.49-13.43]	13.57 [12.77-14.37]	13.58 [12.82-14.35]	0.3505
Mean Thrombocytes (c/nl) [95% CI]	285.5 [251.1-319.9]	260.3 [211.0-309.6]	227.3 [153.5-301.2]	0.1388

Figure S2. Characteristics of liver resection patients.

Clinical, biochemical and haematological characteristics of study patients according to histological steatosis grade. Groups were compared using Fisher's Exact Test or Kruskal-Wallis Test. p-values were not adjusted for multiple comparisons.

		Digestion	Isolation	Expansion	Differentiation	Diet	Fat-Loading
**Basis**							
EBSS		x					
Advanced DMEM/F12			x	x	x		
DMEM						x	x
**Supplements**							
Collagenase D	2.5 mg/ml	x					
DNase I	0.1 mg/ml	x					
Penicillin/Streptomycin	1%		x	x	x	x	x
GlutaMAX	1%		x	x	x	x	x
HEPES	10 mM		x	x	x	x	x
B27	1:50		x	x	x		
N2	1:100		x	x	x	x	x
N-Acetylcysteine	1 mM		x	x	x	x	x
R-spondin 1	1 µg/ml		x	x			
Nicotinamide	10 mM		x	x			
[Leu^{15}]-Gastrin I	10 nM		x	x	x	x	x
EGF	50 ng/ml		x	x	x	x	x
FGF-10	100 ng/ml		x	x			
HGF	25 ng/ml		x	x	x	x	x
Forskolin	10 µM		x	x			
A83-01	5 µM		x	x			
	0.5 µM				x	x	x
Noggin	25 ng/ml		x				
Wnt3a	50 ng/ml		x				
ROCK inhibitor	10 µM		x	until day 6			
DAPT	10 µM				x	x	x
Dexamethasone	3 µM				x	x	x
FGF-19	100 ng/ml				x	x	x
BMP7	25 ng/ml				x	x	x
BSA	1%					x	x
Palmitic Acid:Oleic Acid	1:2 – 2 mM						x

Figure S3. Composition of media used for culturing primary human liver organoids.

a

	488 Excitation					633 Excitation			405 Excitation	
	FL 1<br>AF488	FL 2<br>PE	FL 3<br>ECD	FL 4<br>PC5.5	FL 5<br>PE-Cy7	FL 6<br>AF647	FL 7<br>APC-AF700	FL 8<br>APC-AF750	FL 9<br>BV421	FL 10<br>Aqua
**Antigen**	**IFN-γ**	**IL-22**	**CD8**	**CD56**	**IL-17A**	**IL-4**	**CD4**	**CD3**	**iNKT**	**L/D**
**Clone name**	4S.B3	2G12A41	B9.11	N901	BL168	8D4-8	13BB.2	UCHT1	6B11	Life/dead 506
**Isotype**	IgG1, Mouse	IgG2a, Mouse	IgG1, Mouse	IgG1, Mouse	IgG1, Mouse	IgG1, Mouse	IgG1, Mouse	IgG1, Mouse	IgG1, Mouse	
**Amount (µl)**	2,5 µl	2,5 µl	2,5 µl	2,5 µl	2,5 µl	2,5 µl	5 µl	4 µl	2,5 µl	1 µl
**Supplier**	Biolegend	Biolegend	BC	BC	Biolegend	Biolegend	BC	BC	Biolegend	Invitrogen
**Cat. #**	502515	366704	B08467	B49189	512315	500712	B10824	A94680	342916	65-0866-14

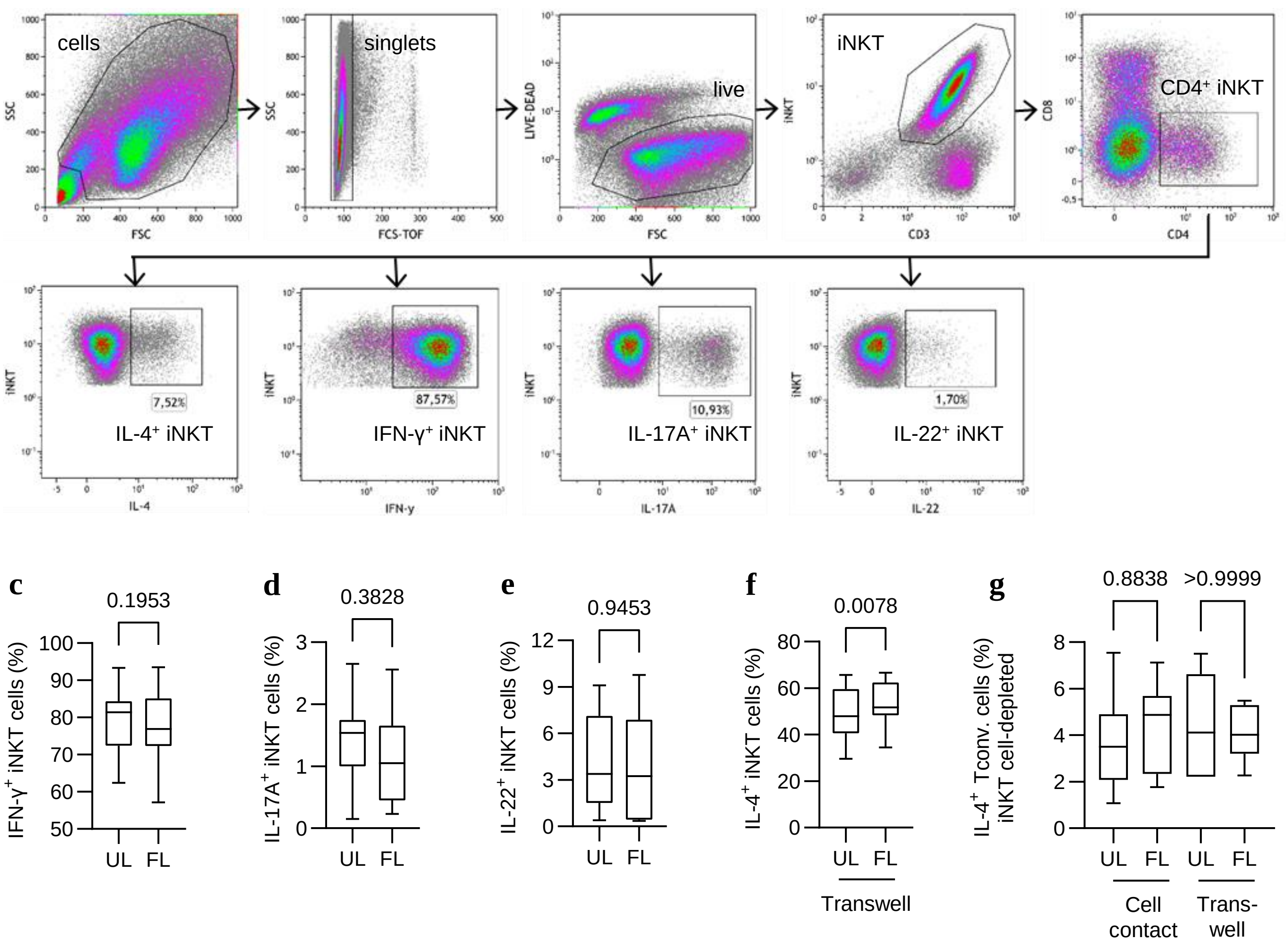

Figure S4. Phenotyping of human iNKT cells after coculture with HepaRG cells.

(A) Optimized flow cytometry panel for assessing human iNKT cell differentiation following *in vitro* expansion.

(B) Flow cytometry gating strategy to determine the frequency of cytokine-expressing CD4⁺ iNKT cells.

(C) Frequency of IFN-γ⁺ iNKT cells after a 7-day expansion in the presence of fat-loaded (FL)- or unloaded (UL)-HepaRG cells [n=8; Wilcoxon matched-pairs signed rank test].

(D) Frequency of IL-17A⁺ iNKT cells after a 7-day expansion in the presence of fat-loaded (FL)- or unloaded (UL)-HepaRG cells [n=8; Wilcoxon matched-pairs signed rank test].

(E) Frequency of IL-22⁺ iNKT cells after a 7-day expansion in the presence of fat-loaded (FL)- or unloaded (UL)-HepaRG cells [n=8; Wilcoxon matched-pairs signed rank test].

(F) Frequency of IL-4⁺ iNKT cells after a 7-day expansion in indirect cocultures with fat-loaded (FL)- or unloaded (UL)-HepaRG cells [n=8;Wilcoxon matched-pairs signed rank test].

(G) Consistent with HepaRG cells specifically suppressing IL-4⁺ iNKT cells, no effect was observed of FL- or UL-HepaRG cells on IL-4 production by conventional T cells [n=7; Friedman test with Dunn's multiple comparisons test].

a

	488 Excitation	405 Excitation
	FL 5<br>Pe-Vio770	FL 10<br>Aqua
**Antigen**	**CD46**	**L/D**
**Clone name**	REA312	life/dead 506
**Isotype**	rhIgG1	
**Amount (µl)**	5	0,5 µl
**Supplier**	Miltenyi Biotec	Invitrogen
**Cat. #**	130-104-510	65-0866-14

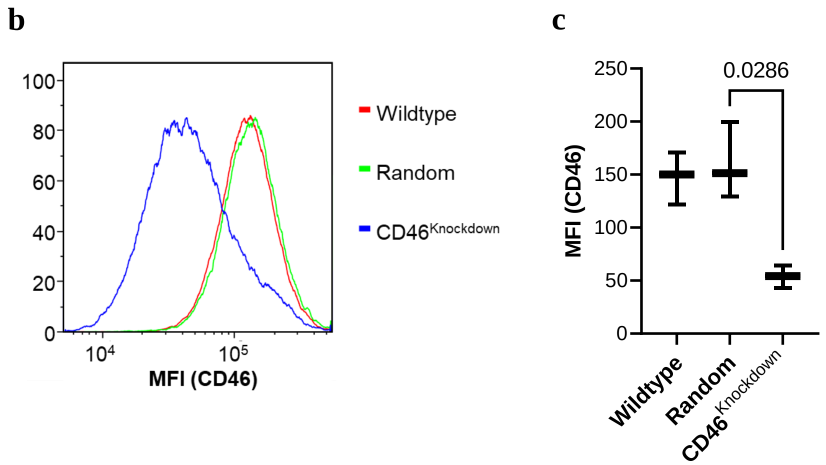

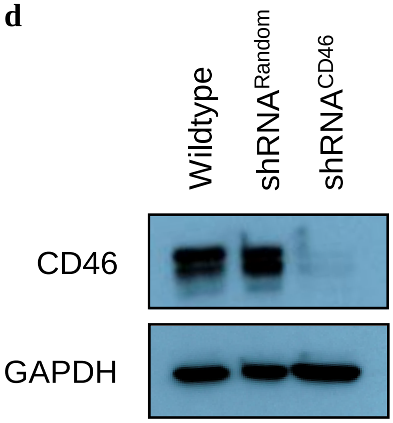

Figure S5. Technical controls for shRNA-silencing of CD46 in HepaRG cells.

(A) Flow cytometry panel for quantifying cell-surface expression of CD46 in HepaRG cells.

(B) Representative flow cytometry results showing down-regulation of cell-surface CD46 expression in HepaRG cells transfected with CD46-shRNA, but not a random shRNA control.

(C) Mean Fluorescence Intensity (MFI) of CD46 expression on unloaded HepaRG cells that were stably transfected with either random shRNA or CD46-knockdown shRNA. Untransfected HepaRG cells are taken as controls. [n=3 independent experiments; Friedman test with Dunn's test for multiple comparisons].

(D) Western blot of CD46 expression showing down-regulation in HepaRG cells transfected with CD46-shRNA, but not a random shRNA control.

Title	Cell-based Assay to Quantify sCD46
Purpose	This document describes a method to measure sCD46 in human serum, plasma and cell culture supernatants

Section	Contents
1.	Abbreviations
2.	Reagents
3.	Preparation of reagents
4.	Protocol for sample preparation

1. ABBREVIATIONS

Abbreviation	Definition
CSB	Cell staining buffer
DPBS	Dulbecco's phosphate buffered saline
RPMI	Roswell Park Memorial Institute

2. REAGENTS

Reagent	Cat. #	Supplier
Advanced RPMI 1640 Medium	12633020	Gibco
CD46 Antibody, anti-human, PE, REAfinity™	130-104-508	Miltenyi Biotec
Cell Staining Buffer	420201	BioLegend
DPBS, no calcium, no magnesium	14190-094	Gibco
FcR blocking reagent, human	130-059-901	Miltenyi Biotec
Fetal calf serum	10270106	Life Technologies
Penicillin/streptomycin	P0781	Sigma-Aldrich
Recombinant Human CD46 Fc Chimera	10257-CD	R&D systems
ViaKrome 808 Fixable Viability Dye	420201	Beckman Coulter

3. PREPARATION OF REAGENTS

3.1 Preparation of MOLT-4 medium

Action	✓
Add 100 µl of fetal calf serum and 10 µl of penicillin/streptomycin per 1 ml RPMI medium	□

3.2 Preparation of the diluted α-CD46-PE

Action	✓
Dilute 0.15 µl α-CD46-PE mAb with 19.85 µl CSB per sample	□

4. PROTOCOL

Step	Action	✓
1	Culture MOLT-4 cells in T75 flasks at 37°C at a starting density of 4×10^5 cells/ml in MOLT-4 medium, performing a complete medium change every 3-4 days: Do not exceed a density of 2 x 10^6 cells/ml.	☐
2	On the day of sCD46 measurement, harvest 3 x 10^5 MOLT-4 cells per sample.	☐
3	Wash the cells with 15 ml DPBS.	☐
4	Resuspend the cells at 4 x 10^6 cells/ml in CSB.	☐
5	Add 0.5 µl ViaKrome 808 Fixable Viability Dye per 10^6 cells.	☐
6	Incubate for 30 min at 4°C in the dark.	☐
7	Meanwhile, prepare a dilution series of chimeric CD46-Fc in a 96-well staining plate starting with 1 µl of a stock concentration of 0.5 µg/µl added to 119 µl of CSB.	☐
8	Perform a serial dilution by transferring 60 µl from one well to the next well containing 60 µl cell staining buffer until 12 standards are prepared; discard 60 µl of the last standard.	☐
9	Add 60 µl of cell culture supernatant, serum or plasma to the 96-well staining plate according to the experimental layout.	☐
10	Add 20 µl of the diluted α-CD46-PE to every sample-containing well.	☐
11	Incubate for 30 min at 4°C in the dark.	☐
12	Meanwhile, wash the MOLT-4 cells with 15 ml CSB.	☐
13	Resuspend the cells in 20 µl CSB per well containing 10% human FcR block.	☐
14	Incubate for 15 min at 4°C in the dark.	☐
15	Add 20 µl of the cell suspension to every well.	☐
16	Incubate for 55 min at 4°C in the dark.	☐
17	Wash the wells with 200 µl CSB twice.	☐
18	Resuspend the cells in 100 µl CSB and analyse the samples by flow cytometer	☐

Figure S6. Cell-based Assay to Quantify sCD46 (Standard Operating Procedure)

a

Oligo[1]	Target	Sequence (5′–3′)	Modification	Length[b]	Tm (°C)[3]
Fw	Exon 6, CD46	TGACAGTAACAGTACTTGGGA			57.3
Rev1	Exon 12/13, CD46	ATCAGTTAGGTATGTGCCTTTC		289/244[2]	58.7
Rev2	Exon 12/14, CD46	ACCATCTGCTTTCCCTTTC		286/241	60.7
Probe	BC, CD46	CCAAAGTGTCTTAAAGTGTCGA CTTCTTCCACTAC	5′-ROX<br>3′-BHQ2		72.0
Probe	C, CD46	AGTGTCTTAAAGGTCCTAGGCC TACTTACAAGC	5′-ROX<br>3′-BHQ2		69.7
Comp	Exon 8/9, CD46	GTGCCTCAGGTCCTAGGCCTA CTTAC	3′-phosphate		68.6

1 Fw, forward primer; Rev, reverse primer; Probe, probe labeled with fluorescent dye and quencher; and Comp, complementary competitor oligonucleotide.
2 Length of amplified BC/C isoform PCR product using exon 6 forward primer.
3 Predicted Tm values.

b

	C1	C2	BC1	BC2	final concentration
Primer_Fw (exon 6)	x	x	x	x	500 nM
Primer_Rev1 (exon 12/13)	x		x		500 nM
Primer_Rev2 (exon 12/14)		x		x	500 nM
Probe_C (exon 6-8)	x	x			175 nM
Probe_BC (exon 6-9)			x	x	175 nM
Comp (exon 8-9)	x	x	x	x	3500 nM

c

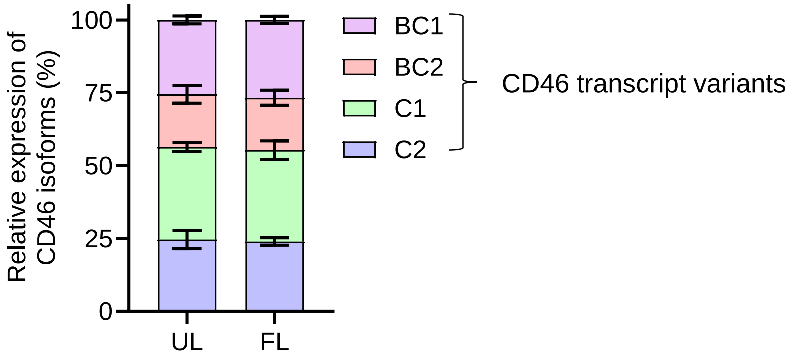

Figure S7. CD46 mRNA is not differentially expressed in fat-loaded and unloaded HepaRG cells.

(A) Primers and probes used for Taqman RT-PCR to determine the relative abundance of the four known CD46 transcript variants. Method adapted from Hansen, A.S. *et al.* (2016). Sci Rep 6, 35406. 10.1038/srep35406.

(B) Transcript variant-specific RT-PCR conditions

(C) No difference was observed in the relative expression of CD46 transcript variants in iNKT cells after coculture with unloaded (UL)- or fat-loaded (FL)-HepaRG cells [n=3].

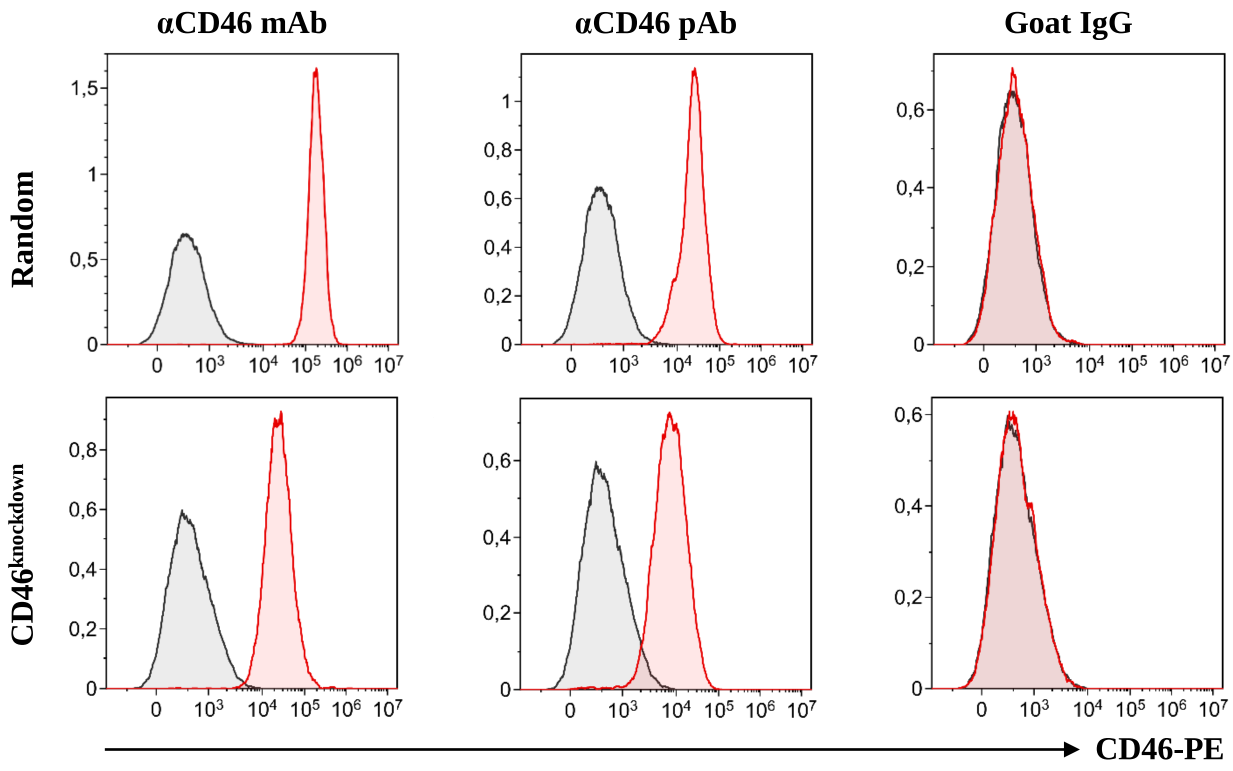

Figure S8. Technical controls for the binding capacity of the polyclonal CD46 antibody.

Representative flow cytometry results showing cell-surface CD46 expression of CD46^{knockdown} and random shRNA control HepaRG cells stained with αCD46-PE mAb (130-104-508, Miltenyi), αCD46 pAb (AF2005, R&D) conjugated with αgoat-PE (F0107, R&D) and goat IgG control (AB-108-C, R&D) conjugated with αgoat-PE. Cells stained with αgoat-PE were used as the negative control.

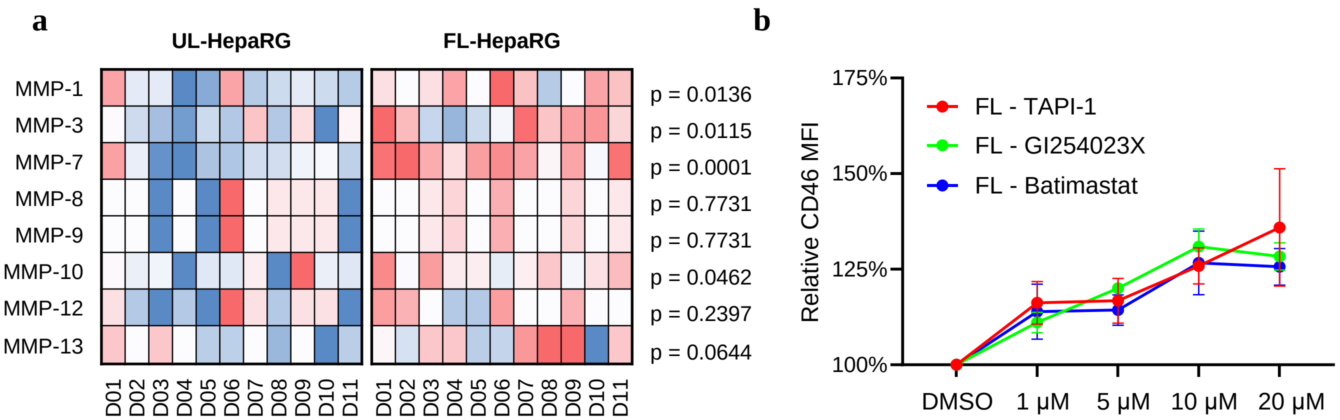

Figure S9. Fat-loading of HepaRG cells induces matrix metalloproteases that degrade CD46.

(A) Heatmap showing standardized concentrations of 8 MMPs in the supernatant of UL- or FL-HepaRG cells [n = 11; 2-tailed BH-corrected t-test].

(B) Relative change in isotype-corrected MFI of CD46 expression in FL-HepaRG cells treated over a range of concentrations with three MMP inhibitors: TAPI-1, GI254023X and batimastat. [n = 3; mean ± SD].

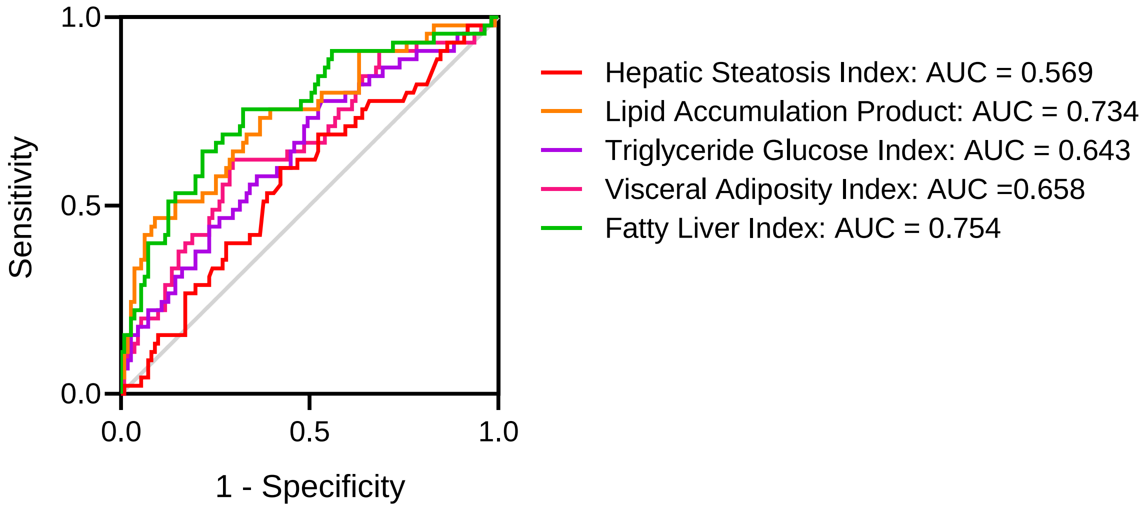

Figure S10. Performance of published clinical scores to predict hepatic steatosis.

ROC curves comparing the performance of 5 composite clinical scores as predictors of hepatic steatosis in n=156 prospective living liver transplant donors. Consistent with published meta-analyses, the most discriminatory score was Fatty Liver Index (FLI) [Contreras, D. *et al*. Ann. Hepatol., 2023; 28(1):100873].

a

Validation of sCD46				
Metric	2.5%	50%	97.5%	Single threshold
threshold	26.91	26.91	26.91	26.91
specificity	0.730	0.824	0.905	0.824
sensitivity	0.533	0.700	0.867	0.700
accuracy	0.712	0.788	0.865	0.788
tn	54	61	67	61
tp	16	21	26	21
fn	4	9	14	9
fp	7	13	20	13
npv	0.809	0.871	0.937	0.871
ppv	0.500	0.622	0.759	0.618
1-specificity	0.095	0.176	0.270	0.176
1-sensitivity	0.133	0.300	0.467	0.300
1-accuracy	0.135	0.212	0.288	0.212
1-npv	0.063	0.129	0.191	0.129
1-ppv	0.241	0.378	0.500	0.382
auc	0.767	0.847	0.915	0.845

b

Validation of Fatty Liver Index				
Metric	2.5%	50%	97.5%	Single threshold
threshold	39.52	47.85	63.31	54.35
specificity	0.568	0.716	0.838	0.743
sensitivity	0.567	0.733	0.867	0.667
accuracy	0.615	0.721	0.808	0.721
tn	42	53	62	55
tp	17	22	26	20
fn	4	8	13	10
fp	12	21	32	19
npv	0.806	0.867	0.933	0.846
ppv	0.404	0.510	0.639	0.513
1-specificity	0.162	0.284	0.432	0.257
1-sensitivity	0.133	0.267	0.433	0.333
1-accuracy	0.192	0.279	0.385	0.279
1-npv	0.067	0.133	0.194	0.154
1-ppv	0.361	0.490	0.596	0.487
auc	0.609	0.730	0.837	0.726

Figure S11. Performance metrics for sCD46 and Fatty Liver Index in predicting steatosis

(A) Performance metrics for sCD46 as a predictor of no steatosis versus steatosis assessed by ultrasonography.

(B) Performance metrics for Fatty Liver Index as a predictor of no steatosis versus steatosis assessed by ultrasonography.

a

Training Set - No Fixed Threshold				
Metric	2.5%	50%	97.5%	Single threshold
threshold	17.31	25.71	35.34	26.19
specificity	0.542	0.750	0.917	0.750
sensitivity	0.571	0.762	0.952	0.714
accuracy	0.622	0.756	0.867	0.733
tn	13	18	22	18
tp	12	16	20	15
fn	1	5	9	6
fp	2	6	11	6
npv	0.667	0.783	0.929	0.750
ppv	0.588	0.720	0.875	0.714
1-specificity	0.083	0.250	0.458	0.250
1-sensitivity	0.048	0.238	0.429	0.286
1-accuracy	0.133	0.244	0.378	0.267
1-npv	0.071	0.217	0.333	0.250
1-ppv	0.125	0.280	0.412	0.286
auc	0.633	0.778	0.897	0.774

b

Training Set - Threshold = 26.19 ng/ml				
Metric	2.5%	50%	97.5%	Single threshold
threshold	26.19	26.19	26.19	26.19
specificity	0.583	0.750	0.917	0.750
sensitivity	0.524	0.714	0.905	0.714
accuracy	0.600	0.733	0.867	0.733
tn	14	18	22	18
tp	11	15	19	15
fn	2	6	10	6
fp	2	6	10	6
npv	0.621	0.750	0.900	0.750
ppv	0.565	0.714	0.882	0.714
1-specificity	0.083	0.250	0.417	0.250
1-sensitivity	0.095	0.286	0.476	0.286
1-accuracy	0.133	0.267	0.400	0.267
1-npv	0.100	0.250	0.379	0.250
1-ppv	0.118	0.286	0.435	0.286
auc	0.633	0.778	0.897	0.774

c

Validation Set - Threshold = 26.19 ng/ml				
Metric	2.5%	50%	97.5%	Single threshold
threshold	26.19	26.19	26.19	26.19
specificity	0.417	0.625	0.792	0.625
sensitivity	0.364	0.591	0.773	0.591
accuracy	0.478	0.609	0.739	0.609
tn	10	15	19	15
tp	8	13	17	13
fn	5	9	14	9
fp	5	9	14	9
npv	0.500	0.625	0.778	0.625
ppv	0.444	0.591	0.750	0.591
1-specificity	0.208	0.375	0.583	0.375
1-sensitivity	0.227	0.409	0.636	0.409
1-accuracy	0.261	0.391	0.522	0.391
1-npv	0.222	0.375	0.500	0.375
1-ppv	0.250	0.409	0.556	0.409
auc	0.553	0.710	0.856	0.710

Figure S12. Performance metrics for sCD46 in predicting histological steatosis grade 0 versus ≥ 1

(A) Performance metrics for sCD46 as a predictor of no steatosis versus steatosis assessed by histology in the Training Set with no fixed threshold.

(B) Performance metrics for sCD46 as a predictor of no steatosis versus steatosis assessed by histology in the Training Set with a threshold = 26.19 ng/ml

(C) Performance metrics for sCD46 as a predictor of no steatosis versus steatosis assessed by histology in the Validation Set with a threshold = 26.19 ng/ml

a

Training Set - No Fixed Threshold				
Metric	2.5%	50%	97.5%	Single threshold
threshold	33.90	43.54	58.38	45.55
specificity	0.667	0.933	1.000	0.933
sensitivity	0.833	1.000	1.000	0.833
accuracy	0.762	0.905	1.000	0.905
tn	10	14	15	14
tp	5	6	6	5
fn	0	0	1	1
fp	0	1	5	1
npv	0.923	1.000	1.000	0.933
ppv	0.545	0.833	1.000	0.833
1-specificity	0.000	0.067	0.333	0.067
1-sensitivity	0.000	0.000	0.167	0.167
1-accuracy	0.000	0.095	0.238	0.095
1-npv	0.000	0.000	0.077	0.067
1-ppv	0.000	0.167	0.455	0.167
auc	0.822	0.956	1.000	0.944

b

Training Set - Threshold = 45.55 ng/ml				
Metric	2.5%	50%	97.5%	Single threshold
threshold	45.55	45.55	45.55	45.55
specificity	0.800	0.933	1.000	0.933
sensitivity	0.500	0.833	1.000	0.833
accuracy	0.762	0.905	1.000	0.905
tn	12	14	15	14
tp	3	5	6	5
fn	0	1	3	1
fp	0	1	3	1
npv	0.824	0.933	1.000	0.933
ppv	0.571	0.833	1.000	0.833
1-specificity	0.000	0.067	0.200	0.067
1-sensitivity	0.000	0.167	0.500	0.167
1-accuracy	0.000	0.095	0.238	0.095
1-npv	0.000	0.067	0.176	0.067
1-ppv	0.000	0.167	0.429	0.167
auc	0.822	0.956	1.000	0.944

c

Validation Set - Threshold = 45.55 ng/ml				
Metric	2.5%	50%	97.5%	Single threshold
threshold	45.55	45.55	45.55	45.55
specificity	1.000	1.000	1.000	1.000
sensitivity	0.500	0.833	1.000	0.833
accuracy	0.864	0.955	1.000	0.955
tn	16	16	16	16
tp	3	5	6	5
fn	0	1	3	1
fp	0	0	0	0
npv	0.842	0.941	1.000	0.941
ppv	1.000	1.000	1.000	1.000
1-specificity	0.000	0.000	0.000	0.000
1-sensitivity	0.000	0.167	0.500	0.167
1-accuracy	0.000	0.045	0.136	0.045
1-npv	0.000	0.059	0.158	0.059
1-ppv	0.000	0.000	0.000	0.000
auc	0.844	0.969	1.000	0.958

Figure S13. Performance metrics for sCD46 in predicting histological steatosis grade 1 versus ≥ 2

(A) Performance metrics for sCD46 as a predictor of grade 1 steatosis versus grade 2-3 steatosis assessed by histology in the Training Set with no fixed threshold.

(B) Performance metrics for sCD46 as a predictor of grade 1 steatosis versus grade 2-3 steatosis assessed by histology in the Training Set with a threshold = 45.55 ng/ml

(C) Performance metrics for sCD46 as a predictor of grade 1 steatosis versus grade 2-3 steatosis assessed by histology in the Validation Set with a threshold = 45.55 ng/ml

Validation Set - Threshold = 45.55 ng/ml				
Metric	2.5%	50%	97.5%	Single threshold
threshold	45.55	45.55	45.55	45.55
specificity	1.000	1.000	1.000	1.000
sensitivity	0.500	0.833	1.000	0.833
accuracy	0.935	0.978	1.000	0.978
tn	40	40	40	40
tp	3	5	6	5
fn	0	1	3	1
fp	0	0	0	0
npv	0.930	0.976	1.000	0.976
ppv	1.000	1.000	1.000	1.000
1-specificity	0.000	0.000	0.000	0.000
1-sensitivity	0.000	0.167	0.500	0.167
1-accuracy	0.000	0.022	0.065	0.022
1-npv	0.000	0.024	0.070	0.024
1-ppv	0.000	0.000	0.000	0.000
auc	0.913	0.979	1.000	0.975

Figure S14. Performance metrics for sCD46 in predicting histological steatosis grade ≤ 1 versus ≥ 2

Performance metrics for sCD46 as a predictor of grade 0-1 steatosis versus grade 2-3 steatosis assessed by histology in the Validation Set with a threshold = 45.55 ng/ml
